# Phage-Derived Endolysins Targeting *Cutibacterium acnes*: A Scoping Review

**DOI:** 10.3390/antibiotics15070697

**Published:** 2026-07-17

**Authors:** Magdalena Sołtys, Rafał Kuczma, Katarzyna Jermakow, Anna Erkiert-Polguj

**Affiliations:** 1 Students’ Scientific Association of Cosmetology, Department of Cosmetology and Aesthetic Dermatology, Faculty of Pharmacy, Medical University of Lodz, Muszyńskiego 1, 91-151 Łódź, Poland; 2 Students’ Scientific Association of Clinical Microbiology, Department of Microbiology, Faculty of Medicine, Wroclaw Medical University, Chałubińskiego 4, 50-368 Wrocław, Poland; rafal.kuczma@student.umw.edu.pl; 3 Department of Microbiology, Faculty of Medicine, Wroclaw Medical University, Chałubińskiego 4, 50-368 Wrocław, Poland; katarzyna.jermakow@umw.edu.pl; 4 Department of Cosmetology and Aesthetic Dermatology, Faculty of Pharmacy, Medical University of Lodz, Muszyńskiego 1, 91-151 Łódź, Poland; anna.erkiert-polguj@umed.lodz.pl

**Keywords:** acne vulgaris, *Cutibacterium acnes*, endolysins, bacteriophages

## Abstract

**Background**: Acne vulgaris is a chronic inflammatory disorder of the pilosebaceous unit in which *Cutibacterium acnes* plays an important role in disease pathogenesis. Rising antimicrobial resistance has intensified interest in selective antimicrobial strategies for acne vulgaris. Bacteriophage-derived endolysins may offer targeted antibacterial activity while potentially sparing the skin microbiota. This scoping review aimed to map current evidence regarding endolysins targeting *C. acnes* and to evaluate their antibacterial activity, selectivity, safety, formulation challenges, and translational potential. **Methods:** This scoping review was conducted in accordance with PRISMA-ScR guidelines. Searches on PubMed, Scopus, Embase, Web of Science, and Google Scholar were performed in February 2026. Experimental studies, academic theses, and patent documents investigating phage-derived endolysins active against *C. acnes* were included and narratively synthesised. **Results:** Five peer-reviewed studies, three academic theses, and seven patent documents were included. Most available evidence originated from in vitro studies, with only one short-term clinical investigation identified. Endolysins and engineered derivatives demonstrated rapid bactericidal activity against *C. acnes*, including multidrug-resistant isolates, while several constructs exhibited minimal activity against selected commensal skin bacteria. Lysin-derived peptides retained activity under physiologically relevant pH and temperature conditions and in the presence of retinoic acid. Major translational challenges included limited stability of enzyme formulations, insufficient pilosebaceous unit penetration data, lack of anti-biofilm evidence and unstandardised microbiome assessment methods. **Conclusions:** Phage-derived endolysins represent a promising targeted therapeutic strategy for acne vulgaris. However, current evidence remains predominantly preclinical, and substantial translational barriers related to delivery, stability, safety, anti-biofilm efficacy, microbiome homeostasis, and clinical validation must be addressed before routine dermatological application.

## 1. Introduction

Acne vulgaris is a common, chronic inflammatory disease of the pilosebaceous unit. This condition significantly impairs patients’ quality of life, and recent data indicate a steady increase in its prevalence. The multifactorial aetiopathogenesis of acne involves four key processes: sebum overproduction, abnormal follicular keratinisation, excessive proliferation of *Cutibacterium acnes*, and inflammatory mechanisms [1,2].

The identification of distinct *C. acnes* phylotypes has reshaped the current understanding of this species’ role in acne development, indicating that the loss of phylogenetic diversity, rather than the mere presence of *C. acnes*, is the crucial factor driving acne lesions. Available evidence suggests that the development of inflammatory acne is associated with the dominance of the IA_1_ phylotype, whereas commensal and potentially protective roles have been attributed to phylotype II [3,4].

Reported mechanisms by which *C. acnes* contributes to acne pathogenesis include the stimulation of sebum secretion, initiation of comedogenesis via the release of free fatty acids and squalene oxidation, modulation of keratinocyte adhesion and proliferation mediated through the IGF-1 signalling pathway and biofilm formation [5,6,7]. The presence of a follicular biofilm limits the efficacy of conventional topical therapies and represents a significant therapeutic barrier [8].

Current guidelines from American and European dermatological societies (the American Academy of Dermatology, 2024, and the European Dermatology Forum, 2025) emphasise the need for an individualised approach to acne vulgaris treatment and the reduction of antibiotic exposure within antimicrobial stewardship strategies, while promoting therapies that preserve the skin microbiota [9,10,11].

These recommendations are driven by a significant increase in antimicrobial resistance among *C. acnes* strains, with global resistance rates reaching 29–37% for erythromycin and 22–31% for clindamycin [12,13], and even exceeding 90% against macrolides and clindamycin in certain regions [14,15], thereby substantially limiting the efficacy of conventional antibiotic therapy for acne.

Antibiotic therapy for acne can induce persistent dysbiosis, manifested by the elimination of commensal species and the impairment of the skin microbiota’s immunomodulatory role. These consequences, induced even by short-term treatment, lead not only to the selection of resistant strains but also to systemic effects on microbial ecosystems beyond the skin [16,17,18].

Current recommendations discourage topical antibiotic monotherapy and advise shortening the duration of systemic therapy, reflecting a global effort to minimise dysbiosis and the selection of resistant strains. Consequently, there is a growing need for targeted therapeutic solutions with a narrower spectrum of activity and greater selectivity towards pathogenic *C. acnes* phylotypes [9,10,11].

In light of these challenges, current biomedical research is increasingly focused on bioprospecting (also known as biodiversity prospecting), which involves the exploration and analysis of biological resources (including organisms and natural substances) to discover innovative therapeutic solutions [19]. In this context, endolysins—bacteriophage-derived lytic enzymes termed enzybiotics—attract particular attention. In contrast to conventional antibiotics used in acne therapy, which typically disrupt key bacterial metabolic processes or protein synthesis, endolysins act through the hydrolysis of peptidoglycan bonds within the bacterial cell wall, leading to rapid bacterial cell lysis [20]. Due to the highly conserved nature of structural components in the Gram-positive bacterial cell wall, resistance to endolysins is generally considered less likely to emerge than resistance to conventional antibiotics. The modular domain architecture of these enzymes enables the rational engineering of molecules with enhanced specificity towards selected bacterial targets, aligning with the concept of microbiome-sparing therapy [21].

Despite growing interest in endolysins as an alternative to antibiotics, available evidence regarding their activity against *C. acnes*, specificity of action, safety, and translational potential remains limited and fragmented.

This scoping review aimed to systematically map the available scientific evidence regarding phage-derived endolysins targeting *C. acnes*, and to critically analyse experimental studies evaluating the antimicrobial activity, specificity, formulation challenges, and potential for topical therapy of these enzymes and their derivatives.

## 2. Results—Evidence Mapping

### 2.1. Empirical Data from Peer-Reviewed Scientific Publications

#### 2.1.1. Characteristics of the Included Studies

Five studies published between 2023 and 2025 were analysed [22,23,24,25,26]. These studies originated from research centres in Iraq [25], South Korea [22,24], Greece [23], and the United States [26].

Four studies were experimental in vitro investigations [22,23,24,26], while one was a clinical trial evaluating the efficacy of topical therapy alongside microbiological analysis [25].

#### 2.1.2. Mechanisms of Action and Antimicrobial Activity In Vitro

The spectrum of analysed protein constructs encompassed various engineered variants of phage endolysins, including full-length recombinant *C. acnes* phage endolysins (PaAmi1, CAP10-3 endolysin) [22,23], isolated N-terminal catalytic domains (N1 domains) of these enzymes [24], artilysin-type fusion proteins (PA1PaAmi1) [23], and synthetic cationic peptides derived from endolysins (peptide P156) [26]. Specifically, peptide P156 is an engineered derivative of the C-terminal cationic region of the PlyPi01 lysin. The peptide was structurally modified through the addition of arginine residues to enhance its broad-spectrum bactericidal efficacy [26].

The recombinant endolysins targeting *C. acnes* were generally described as modular proteins composed of an N-terminal enzymatically active domain (EAD) and a C-terminal cell wall-binding domain (CBD) [23,24]. These full-length recombinant endolysins were predominantly classified as N-acetylmuramoyl-L-alanine amidase type 2 (NALAA-2; EC 3.5.1.28) [22,23,24]. In contrast, PlyPi01, from which peptide P156 was derived, was identified in a bacteriophage infecting the Gram-negative anaerobe *Prevotella intermedia* and was reported to contain a predicted globular muramidase domain [26].

Available bioinformatic analyses further classified the N-terminal EAD of PaAmi1 within the peptidoglycan recognition protein (PGRP) superfamily and identified a conserved NALAA-2 catalytic domain [23]. Similarly, CAP10-3 was classified as a PGRP-family protein based on sequence homology [24]. Structural characterisation relied exclusively on in silico approaches, including I-TASSER [23], AlphaFold [24], and SWISS-MODEL [26]. These analyses suggested the presence of conserved catalytic domains and putative C-terminal binding regions in the modular *C. acnes*-targeting endolysins [23,24]. However, experimentally resolved structures have not yet been reported in the mapped literature.

Experimental studies demonstrated that the catalytic activity of these constructs led to a rapid reduction in viable bacterial cell counts, confirming their bactericidal activity [22,23,24]. In vitro models were used to evaluate the lytic and bactericidal activity of endolysins and their derivatives against reference strains and clinical isolates of *C. acnes*. Antimicrobial activity was assessed via turbidimetric measurement of the optical density (OD) of the bacterial biomass suspension [22,23,24], determination of the reduction in colony-forming units (CFUs) in microbiological cultures [22,23,24,26], spot lysis assays [25], and morphological analysis via scanning electron microscopy (SEM) [24].

Biochemical assays demonstrated an increase in the lytic activity of the PaAmi1 endolysin in the presence of zinc ions (5 mM ZnCl_2_) [23]. A comparison of the catalytic domains of the CAP10-3 endolysin indicated higher lytic activity for the isolated N-terminal domain (N1) than for the full-length protein in the OD_600_ reduction assay [24]. In bactericidal kinetics assays, peptide P156 caused a rapid CFU reduction exceeding 3 log_10_ within 1 min and 5 log_10_ within 10 min at a concentration of 25 µg/mL [26]. Full-length recombinant endolysins demonstrated preferential activity against tested *C. acnes* strains, with minimal activity against selected non-target skin-colonising bacteria in the analysed experimental models [23,24], including a lack of effect on *Staphylococcus aureus* strains and negligible effects on *Staphylococcus epidermidis* [24]. Peptide P156 exhibited activity against *S. aureus* strains (both MRSA and MSSA) [26]. Conversely, modified artilysin-type variants were active against enterococci and *Acinetobacter baumannii*, indicating an expanded spectrum of activity [23]. Among the included studies, only Sela et al. explicitly reported phylotype information, evaluating representatives of the IA and IB phylotypes [26], whereas phylotype characterisation was absent from the remaining investigations.

#### 2.1.3. Stability and Safety

The safety profile was evaluated using in vitro cytotoxicity assays and clinical observations during trials.

As demonstrated by Sela et al., peptide P156 maintained high bactericidal activity (>4 log_10_ CFU reduction) across a temperature range of 20–40 °C and a pH range of 5.5–8.0, while exhibiting a gradual decrease in activity under conditions of increased salinity (greater than 50 mM NaCl). Furthermore, the antibacterial effect of the peptide was preserved when evaluated in combination with retinoic acid at concentrations ranging from 0.01% to 0.1%. In in vitro cytotoxicity assays, no haemolytic effect of peptide P156 on human erythrocytes was observed at concentrations up to 256 µg/mL [26].

The study by Hussain et al. demonstrated that the stability of a 1% hydroxyethyl cellulose (HEC)-based endolysin gel formulation was limited to 14–21 days at 25 °C, whereas its activity was preserved for 21 days at 4 °C [25].

Across the mapped literature, safety evaluations were limited to in vitro haemolysis assays [26] and short-term clinical observations of allergic reactions [25]. Data regarding other dermatological safety parameters, such as contact sensitisation, local irritation profiles, or long-term immunogenicity following repeated application, were not reported in any of the included studies.

#### 2.1.4. In Vivo Clinical Outcomes

In the clinical trial conducted by Hussain et al., the investigated active agent was a naturally extracted and purified endolysin from a wild-type *C. acnes* phage. The HEC-based endolysin gel, as well as a mixture gel combining the endolysin with a whole-phage cocktail, was applied for 30 min three times daily for 7 days on a localised 2 cm^2^ skin area. The study reported decolonisation of multidrug-resistant (MDR) *C. acnes* in treated lesions during short-term follow-up, accompanied by a reduction in lesion count and size. No allergic reactions were reported during the treatment period. Furthermore, within a parallel in vitro arm of the same study, the formulation demonstrated lytic efficacy comparable to 2% fusidic acid and 2% mupirocin [25]. The authors did not report the use of a standardised acne severity grading scale and did not provide detailed information regarding participant baseline characteristics and post-treatment follow-up.

Table 1 provides a detailed overview of all endolysins and endolysin-derived constructs identified in the peer-reviewed literature, including their specificity, antimicrobial activity, and selected experimental characteristics.

### 2.2. Academic Theses

Academic theses were analysed as a source of grey literature. Unlike peer-reviewed publications, these documents had not undergone formal external peer review prior to inclusion in this review. The analysis encompassed three academic theses completed at academic centres in the United States and Ireland between 2023 and 2024 [27,28,29].

Similarly to the peer-reviewed publications, these projects were predominantly preclinical in nature and were conducted exclusively using in vitro models.

The studies focused on endolysins with a modular two-domain structure (EAD-CBD), derived primarily from bacteriophages belonging to the genus *Pahexavirus* [27,29].

The characterisation of these enzymes as amidases and their species-specific activity against *C. acnes* were reported, although experimental data evaluating their activity against other skin-colonising microorganisms were scarce [28].

Lytic efficacy was verified using turbidimetric assays, demonstrating dependence on the bacterial growth phase and metal ion concentrations, particularly zinc ions (Zn^2+^) [27].

A significant aspect reported across these studies involved biotechnological challenges, particularly the low solubility of the analysed proteins and their propensity to form inclusion bodies in *Escherichia coli*-based expression systems [27,29].

### 2.3. Patents and Other Technological Data

The evidence derived from patent applications requires cautious interpretation. Unlike peer-reviewed experimental studies, patent claims undergo legal rather than scientific scrutiny, often lacking rigorous statistical validation, standardised methodological descriptions or independent reproducibility. Therefore, data regarding lytic activity and formulation stability extracted from patents were interpreted separately from peer-reviewed experimental evidence.

Seven patent applications published between 2012 and 2026, representing both commercial entities and an academic centre, were included in the analysis [30,31,32,33,34,35,36]. The included applications varied in legal status. The analysis encompassed technologies based on endolysins targeting *C. acnes*, including recombinant protein constructs, their methods of production, and formulation strategies.

These documents described recombinant full-length endolysins [31,33,34,36], their isolated domain variants [35], chimeric constructs [30], and other fusion proteins [32].

The analysed applications presented preclinical data obtained from in vitro models, encompassing the evaluation of the lytic activity of engineered protein constructs; some also incorporated biofilm models [30,31]. Activity was most frequently reported as relative changes in the optical density (OD) of the bacterial suspension or non-standardised ‘percentage lysis’ metrics (often representing a proxy for OD reduction or clearing zone area), rather than the standardised logarithmic reductions typically employed in peer-reviewed microbiological studies. While patent applications by Assaf et al. [30] and Izadjoo et al. [31] claimed anti-biofilm activity based on in vitro models (e.g., polystyrene plate assays), these datasets lacked standardised, quantitative endpoints. Currently, evidence of anti-biofilm efficacy remains restricted to the patent literature, with no in vivo or clinical data available to date.

Some documents described the combination of endolysins with additional ingredients intended to enhance antimicrobial activity (e.g., compounds derived from essential oils) [34], alongside formulation strategies involving non-ionic polymers and modification of formulation parameters, such as pH and electrolyte concentration, to maintain lytic activity under application conditions [33]. It was indicated that enzymatic activity was highest under near-neutral pH conditions and at low electrolyte concentrations (≤50 mM), whereas an increase in salinity was associated with a decrease in activity [33]. The importance of these parameters for preserving enzyme activity in formulations intended for topical application was emphasised.

The proposed applications included both cosmetic and therapeutic uses. The documents described cosmetic products (both leave-on and rinse-off) [30,33,34], topical dermatological formulations, and dressings serving as enzyme carriers [31]. The scope of protection for some patents extended to multi-species applications, including the simultaneous targeting of *C. acnes* and *S. aureus* (including MRSA strains) [35].

## 3. Discussion

### 3.1. Selectivity of Endolysins Against the Skin Microbiome

The skin microbiome supports natural defence against pathogens while playing a vital role in modulating the host immune system. The presence of opportunistic microorganisms underscores the importance of maintaining microbiome homeostasis to preserve the integrity of both the immunological and physical barriers [37,38]. Disruptions to this balance, termed dysbiosis, impair the skin’s barrier function, which is linked to the exacerbation of inflammatory processes [38]. The literature also highlights a correlation between the alleviation of clinical acne symptoms and an increase in alpha diversity, which reflects the degree of microbial diversity within a given environment [39]. Concurrently, contemporary models of pathogenesis account for phylotype-level heterogeneity within the skin microbiome, including the significance of specific, pro-inflammatory *C. acnes* phylotypes and their interactions with commensal microorganisms [4,40].

The impact of conventional anti-acne therapies on the cutaneous ecosystem is variable and determined by the mechanism of action of the active agent used [39,41]. While systemic antibiotic therapy can paradoxically increase alpha diversity by reducing *C. acnes* dominance [39,41], the non-selective mechanism of benzoyl peroxide (BPO) has been linked to a statistically significant reduction in microbial diversity indices [39]. These observations suggest that, beyond antimicrobial efficacy, minimising the impact on the commensal skin microbiota is becoming a crucial aspect of developing novel anti-acne therapies.

In light of the findings of this review, phage-derived endolysins emerge as a potentially more selective therapeutic strategy. This is supported by findings from studies on the recombinant CAP10-3 endolysin, where its N-terminal (N1) domain demonstrated high selectivity towards *C. acnes* in vitro. Importantly, this isolated domain demonstrated superior bactericidal efficacy against *C. acnes* compared to the full-length enzyme. These findings suggest that removal of the CBD does not necessarily impair enzymatic access to the peptidoglycan substrate or compromise species-level selectivity [24]. Meanwhile, the impact of the N1 domain on the population of *S. epidermidis*, a model representative of the physiological epidermal microbiota selected due to its protective barrier functions and high relative abundance on healthy skin [42], remained marginal; a statistically significant reduction in viable cell counts from 9.36 to 8.97 log CFU/mL was observed only at a concentration of 100 µg/mL [24]. However, it must be emphasised that most studies on endolysins against *C. acnes* focus primarily on their lytic activity, frequently overlooking their impact on the commensal skin microbiota. Claims that endolysins are ‘microbiome-sparing’ remain largely extrapolated from narrow in vitro specificity assays rather than robust in vivo microbiome profiling. While these assays suggest that endolysins possess a narrow lytic spectrum, potentially sparing commensal species, they are insufficient to definitively conclude that they preserve complex in vivo skin microbiome homeostasis. Currently, no rigorous longitudinal clinical studies have evaluated whether repeated topical administration of endolysins preserves skin microbiome stability or alters commensal bacterial recolonisation dynamics over time. This significant research gap hinders a comprehensive assessment of their translational potential.

### 3.2. Phage Diversity, Phylotype Selectivity, and Resistance Considerations

Contemporary microbiome-based models of acne pathogenesis emphasise the importance of phylotype-level heterogeneity within *C. acnes* populations, particularly the contribution of pro-inflammatory phylotypes to disease development [1,3]. In this context, an ideal therapeutic model should entail the selective clearance of pathogenic strains while preserving commensal phylotypes, which may exert protective functions in healthy individuals.

The high amino acid sequence conservation of *C. acnes* endolysins, reaching up to 95%, is consistent with reports of broad activity against diverse isolates [43,44]. The narrow genome size range and consistent GC content within the *C. acnes* bacteriophage population indicate low diversity within its gene pool compared to other phage populations [43,45]. This genetic homogeneity may explain the high conservation of endolysin sequences. While this phenomenon may facilitate broad lytic activity against diverse clinical isolates, it simultaneously poses a substantial challenge for designing therapies that exhibit selectivity towards pro-inflammatory *C. acnes* phylotypes.

Crucially, the mapped experimental models predominantly utilised laboratory strains or clinical isolates. While one study explicitly provided phylotype information (Type IA and IB) for the investigated strains [26], none of the included studies systematically compared the lytic activity of these endolysins across distinct pathogenic (e.g., IA_1_) and commensal (e.g., II) phylotypes. Consequently, while models of acne pathogenesis highlight the importance of selectively targeting pro-inflammatory phylotypes, existing empirical evidence does not yet confirm whether these endolysins possess phylotype-specific lytic activity or if they universally lyse all *C. acnes* clades.

The limited diversity of the *C. acnes* phage population, together with the presence of several phage-resistance mechanisms, provides a useful context for considering the distinct therapeutic properties of endolysin-based approaches relative to whole-phage therapy. Several mechanisms contributing to phage resistance have been described in *C. acnes*, including the CRISPR-Cas systems in type II strains, restriction–modification (R-M) systems, and superinfection immunity associated with pseudolysogenic phages [46]. Unlike bacteriophages, endolysins do not depend on bacterial receptor recognition, intracellular replication, or completion of the phage infection cycle, but instead exert their antibacterial activity through direct hydrolysis of highly conserved peptidoglycan structures. This mechanism may reduce susceptibility to several resistance pathways that compromise the efficacy of conventional antibiotics or whole-phage therapy [21,47,48]. However, this assumption remains largely based on mechanistic considerations and observations derived from other bacterial systems rather than direct experimental evidence in *C. acnes*. Moreover, no studies evaluating adaptive responses to prolonged endolysin exposure, including experimental evolution models, were identified in the mapped literature. Consequently, both the long-term evolutionary consequences of endolysin therapy and its potential impact on phylotype-level population dynamics remain insufficiently characterised and warrant further investigation.

### 3.3. The Pilosebaceous Unit as a Pharmacological Barrier for Endolysin Therapy

**Figure 1:**
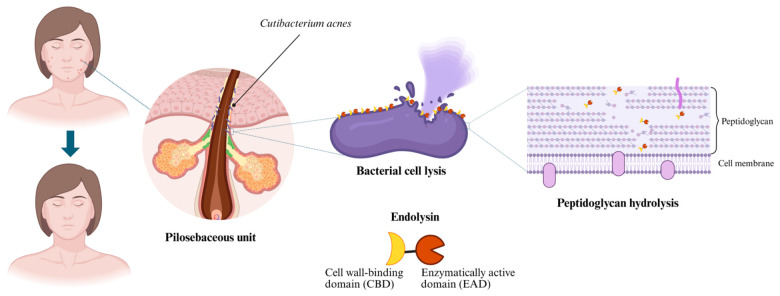
Schematic representation of the mechanism of action of a phage-derived endolysin targeting *Cutibacterium acnes* within the pilosebaceous unit in acne vulgaris. The figure sequentially illustrates: the localisation of *C. acnes* within the hair follicle, the interaction of the endolysin with the bacterial cell wall, bacterial cell lysis, and the molecular mechanism of peptidoglycan hydrolysis leading to cell wall destabilisation. The modular architecture of the endolysin is presented at the bottom, comprising a cell wall-binding domain (CBD) responsible for recognition of the target bacterium and an enzymatically active domain (EAD) that catalyses peptidoglycan degradation. Created in BioRender. Sołtys, M. (2026) https://BioRender.com/hxaefpq.

The pilosebaceous unit (PSU) may be viewed as a distinct pharmacological compartment wherein interactions between the microbiome, biofilm, and the physicochemical properties of sebum influence the local pharmacokinetics and pharmacodynamics of anti-acne therapies, including antimicrobial agents (Figure 1).

The PSU constitutes a specific pharmacokinetic microenvironment wherein the processes of pathological hyperkeratinisation and sebum accumulation create impediments that affect the penetration and retention of therapeutic agents [7,49]. An additional biological challenge is the ability of *C. acnes* to form biofilm structures, whose extracellular polymeric substances (EPS) matrix, composed primarily of polysaccharides, proteins, and eDNA, functions as a biological shield and a physical diffusion barrier, thereby limiting the bioavailability of conventional therapeutics [50,51]. Despite the recognised importance of biofilms in acne pathogenesis, direct experimental evidence regarding the anti-biofilm activity of *C. acnes*-targeting endolysins is currently limited to patent-derived in vitro models, and no peer-reviewed experimental studies evaluating this endpoint have been identified.

Although one included study evaluated a topical HEC gel formulation, none of the identified studies directly investigated the follicular penetration, tissue distribution, or local bioavailability of endolysins within the PSU. Consequently, the optimal delivery strategy for these molecules remains unknown and represents a major translational challenge. Future studies should therefore evaluate follicular penetration and local pharmacokinetics while exploring advanced formulation strategies capable of overcoming the physiological barriers of the human hair follicle.

### 3.4. Physicochemical, Manufacturing, and Safety Determinants of Endolysin Therapy

The therapeutic efficacy of endolysins against *C. acnes* remains dependent on their stability within the complex microenvironment of the skin. Accumulated literature data suggest that physiologically relevant conditions, particularly regarding pH and temperature, maintain the lytic activity of selected endolysins, whereas increased ionic strength may restrict the efficacy of certain constructs. Notably, the lytic activity of certain endolysins, such as PaAmi1, is enhanced in the presence of zinc ions (Zn^2+^). This suggests the possibility of therapeutic synergy, considering the well-established safety profile of zinc and its documented supportive role in acne treatment [52].

Due to their proteinaceous nature, endolysins remain susceptible to environmental factors and proteolytic degradation [53]. In this context, the capacity of the endolysin-derived peptide P156 to maintain high bactericidal activity across a wide temperature range (20–40 °C) supports its further translational evaluation.

Chimeric endolysins, designed through the modification and fusion of functional domains, represent a promising avenue for optimisation. This approach may allow for the generation of constructs with enhanced lytic activity and potentially expanded functionality, such as anti-biofilm properties, compared to their native counterparts. Nonetheless, these constructs can promote protein aggregation in the form of insoluble inclusion bodies within *E. coli* expression systems, which substantially hinders manufacturing scale-up and increases product costs [54]. Ultimately, this manufacturing challenge may increase barriers to market entry for these novel antimicrobial agents, particularly when competing against highly cost-effective generic topical antibiotics in conventional acne management [55]. Selecting the optimal expression host remains a critical determinant of production yield and scalability when transitioning from laboratory to industrial scale. While *E. coli* strains represent the most widely utilised and highly optimised platform for recombinant protein production, the need for extensive endotoxin removal and multi-step downstream purification increases manufacturing complexity and may contribute to higher production costs [53,56].

The formulation stability of endolysins also remains a major translational challenge. For instance, the reported activity of an experimental endolysin gel formulation was maintained for 14–21 days at 25 °C and up to 21 days at 4 °C, which may limit the translational potential of such solutions. Considering that standard stability testing of medicinal products constitutes an integral part of the regulatory approval process and encompasses a period of at least 12 months [57], achieving adequate formulation shelf-life presents a substantial barrier to clinical translation. These limitations simultaneously provide a rationale for the development of novel formulation strategies and endolysin delivery systems.

Another poorly explored aspect concerns the potential immunological consequences of rapid bacterial lysis induced by endolysins. Given the recognised contribution of *C. acnes*-derived cell wall components and other bacterial products to inflammatory signalling pathways involved in acne pathogenesis, the rapid release of bacterial products following endolysin-mediated lysis may theoretically influence local immune responses within the pilosebaceous unit [58,59]. However, this possibility has not been directly investigated for endolysins targeting *C. acnes* and remains insufficiently characterised.

The safety data currently available in the mapped literature are preliminary. Standard dermatological safety parameters, such as contact sensitisation and local irritation, have not been systematically evaluated for *C. acnes* endolysins. Furthermore, there are no data regarding potential long-term immunogenicity, such as the formation of neutralising antibodies following repeated topical application of these recombinant proteins. Comprehensive toxicological and immunological profiling is therefore required to support further clinical development.

### 3.5. Advances in Lytic Protein Engineering and the Potential of Combination Therapy

The limited stability and delivery challenges of endolysins remain major drivers for the development of novel protein engineering strategies and therapeutic formulations. Over the past two decades, numerous phage-derived endolysins and engineered lysin variants have been developed as antimicrobial agents against *S. aureus* [60]. This is partly attributable to the classification of methicillin-resistant *S. aureus* (MRSA) as a priority pathogen by the WHO and CDC [61,62], which has catalysed the creation of innovative topical formulations and delivery strategies that may serve as a benchmark for future *C. acnes*-targeted therapies. These strategies include the fusion of endolysins with cell-penetrating peptides (CPPs), the utilisation of hydrogels and nanoemulgels, encapsulation within liposomes, and the immobilisation of enzymes onto cellulose nanostructures [63].

Contemporary approaches to lytic protein engineering, represented by third-generation lysins, extend beyond bacterial eradication to encompass the optimisation of pharmacokinetic parameters and the modulation of the host inflammatory response [63]. Given the multifactorial and inflammatory nature of acne pathogenesis, this approach provides a rationale for combination therapies. An example of such a strategy is the combination of lytic enzymes with anti-inflammatory substances, such as flavonoids [64], which may facilitate the simultaneous reduction in inflammatory mediators while decreasing bacterial load.

Acne is not classified as an infectious disease; hence, therapeutic strategies aimed solely at reducing *C. acnes* colonisation typically do not achieve complete resolution of lesions [65]. Current standards for the topical treatment of mild-to-moderate acne include fixed-dose combination products containing clindamycin and tretinoin (all-trans retinoic acid) [10]. Historically, clindamycin replaced erythromycin due to the emergence of bacterial resistance; however, a rising prevalence of lincosamide resistance among *C. acnes* strains has been reported [12,66].

Topical retinoids promote the normalisation of follicular keratinisation and enhance the penetration of other topically applied anti-acne agents [67]. This mechanism could theoretically facilitate the delivery of endolysins to follicular niches colonised by *C. acnes*, which is of particular interest in light of data demonstrating that the bactericidal activity of peptide P156 is preserved in the presence of retinoic acid [26]. To the best of our knowledge, this is currently one of the few available reports regarding endolysin stability in the presence of retinoids. The scarcity of data in this area highlights the need for further investigations into formulation stability and potential therapeutic synergy in in vivo models.

### 3.6. Translational Pathway and Regulatory Considerations

One of the earliest examples of endolysin commercialisation is Staphefekt SA.100, a chimeric endolysin introduced to the European market in 2017 by the Dutch company Micreos. This formulation was registered as a Class I medical device and is available in the form of a cetomacrogol-based gel or cream. The enzyme specifically targets *S. aureus*, including MRSA strains, allowing for their selective clearance without significantly disrupting the skin microbiota [68,69].

In a randomised controlled trial involving patients with atopic dermatitis, the safety and tolerability of the formulation were confirmed during 12 weeks of therapy [70]. Furthermore, in a single-patient case study, no changes in MIC values were observed following several months of application; however, the scope of available data does not allow for an assessment of the potential for resistance development during long-term use [69]. Current experience regarding the use of endolysins in dermatoses associated with staphylococcal colonisation may indicate their therapeutic potential, yet the possibility of directly extrapolating these observations to acne therapy remains uncertain. Available clinical data regarding endolysins targeting *C. acnes* remain highly limited and primarily encompass short-term pilot observations.

Caution must also be exercised when directly extrapolating the regulatory classification pathway of the SA.100 endolysin to novel strategies based on lytic enzymes against *C. acnes*. The literature indicates that most endolysin-based therapeutic strategies utilise recombinant DNA technologies and heterologous protein expression, which entail additional requirements regarding manufacturing and safety evaluation. Moreover, the lack of a unified regulatory framework for endolysin applications poses a barrier to clinical translation [71]. Nevertheless, unlike therapies utilising whole bacteriophages, which function as self-replicating biological agents, endolysins are non-replicating protein therapeutics devoid of genetic material. This distinction eliminates the risk of transduction, simplifies the evaluation of selected safety aspects, and allows these enzymes to follow a more conventional translational and regulatory pathway [72].

### 3.7. Limitations

**Figure 2:**
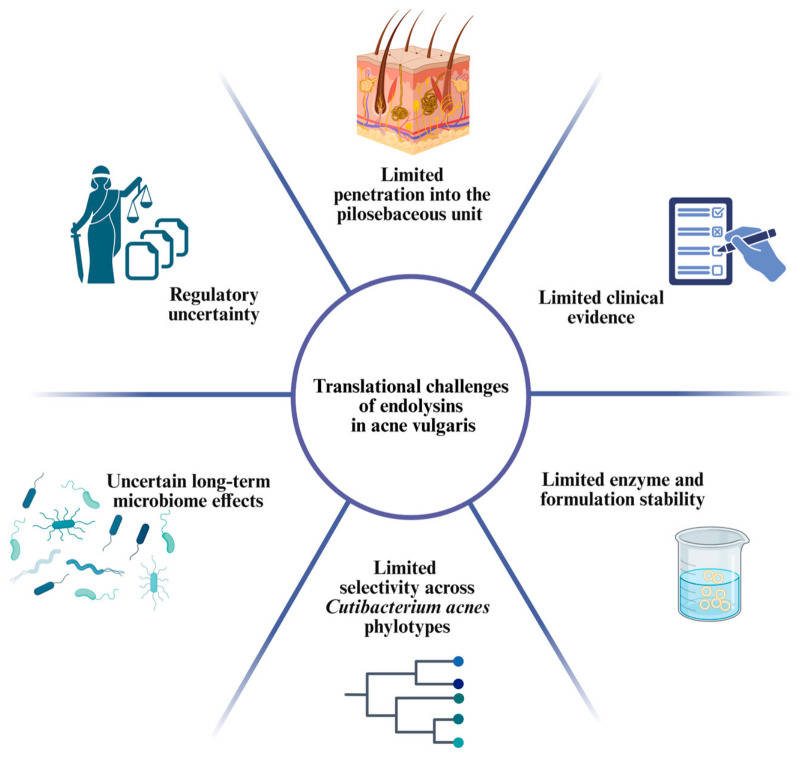
Schematic representation of the primary translational challenges associated with endolysin-based therapy for acne vulgaris. Created in BioRender. Sołtys, M. (2026). https://BioRender.com/p7coexp.

Several limitations of the available evidence should be acknowledged. The majority of the analysed studies were in vitro investigations, and the available clinical evidence is limited to a single short-term study. A considerable proportion of the mapped evidence originated from grey literature sources, including academic theses and patent documents, which differ from peer-reviewed studies in terms of scientific validation and reporting standards. Direct comparison of antimicrobial efficacy across studies was hindered by differences in experimental models and outcome measures. No longitudinal studies assessing the impact of endolysins on skin microbiome composition, diversity, or recolonisation dynamics were identified. Evidence regarding anti-biofilm activity remains restricted to patent-derived in vitro models, with an absence of peer-reviewed data for this endpoint. Likewise, no studies directly investigated the follicular penetration, tissue distribution, or local bioavailability of endolysins within the pilosebaceous unit. The primary barriers hindering the clinical translation of endolysins for acne vulgaris are summarised in Figure 2.

## 4. Materials and Methods

This scoping review was conducted in accordance with the Preferred Reporting Items for Systematic Reviews and Meta-Analyses Extension for Scoping Reviews (PRISMA-ScR) guidelines. The objective of the review was to systematically map the available scientific evidence regarding phage-derived endolysins targeting *C. acnes*, encompassing both experimental studies and patent applications. A completed PRISMA-ScR checklist is provided as Supplementary File S1. The protocol for this scoping review was registered retrospectively on the Open Science Framework (URL: https://osf.io/7jyxa; DOI: 10.17605/OSF.IO/7JYXA, accessed on 12 February 2026).

### 4.1. Information Sources and Search Strategy

A comprehensive literature search was conducted on 12 February 2026 across the following databases: PubMed, Scopus, Embase, Web of Science, and Google Scholar. For PubMed, Scopus, Embase, and Web of Science, the following search strategy was applied, with syntax adapted to the specific requirements of each database: ‘(endolysin OR endolysins OR lysin OR phage-derived OR phage lysin OR bacteriophage lysin OR phage lytic enzyme OR enzybiotic OR cell wall hydrolase OR endolysin-derived peptide OR lysin-derived peptide OR engineered lysin peptide OR cationic lytic peptide OR peptide derived from endolysin OR peptide derived from lysin OR lysin-derived antimicrobial peptide) AND (*Cutibacterium acnes* OR *Propionibacterium acnes* OR *C. acnes*)’. In the Google Scholar database, the advanced search function was used with the following parameters: all words: ‘endolysin’; with at least one of the words: ‘*Cutibacterium acnes*’, ‘*Propionibacterium acnes*’, ‘*C acnes*’; terms appearing anywhere in the text; date restrictions: no time restrictions; include patents: enabled; citations excluded.

No language restrictions were applied during the initial search phase; however, only publications available in English were considered for final analysis. In Google Scholar, results were screened for relevance to retrieve academic theses and patent applications via Google Patents, resulting in the screening of all 537 retrieved records.

Additionally, a manual search of the reference lists of included publications was performed to identify potential supplementary records. The retrieved results were exported in formats compatible with reference management software and imported into the Covidence platform (Veritas Health Innovation, Melbourne, Australia; available at: https://www.covidence.org/ (accessed on 12 February 2026)) for further screening and eligibility assessment. Two independent reviewers performed the study selection and data extraction processes. Any discrepancies were resolved through consensus.

### 4.2. Study Selection Process

The study selection process was conducted using the Covidence platform and comprised three stages: (1) title and abstract screening, (2) full-text assessment, and (3) final inclusion for analysis.

Duplicates were automatically identified by the Covidence system and manually verified during the screening stage.

At the title and abstract screening stage, records potentially related to phage-derived endolysins, including original research articles, academic theses, and patent documents, were considered eligible for further evaluation. Secondary publications (review articles, commentaries, letters to the editor, expert opinions) were excluded at this stage.

**Figure 3:**
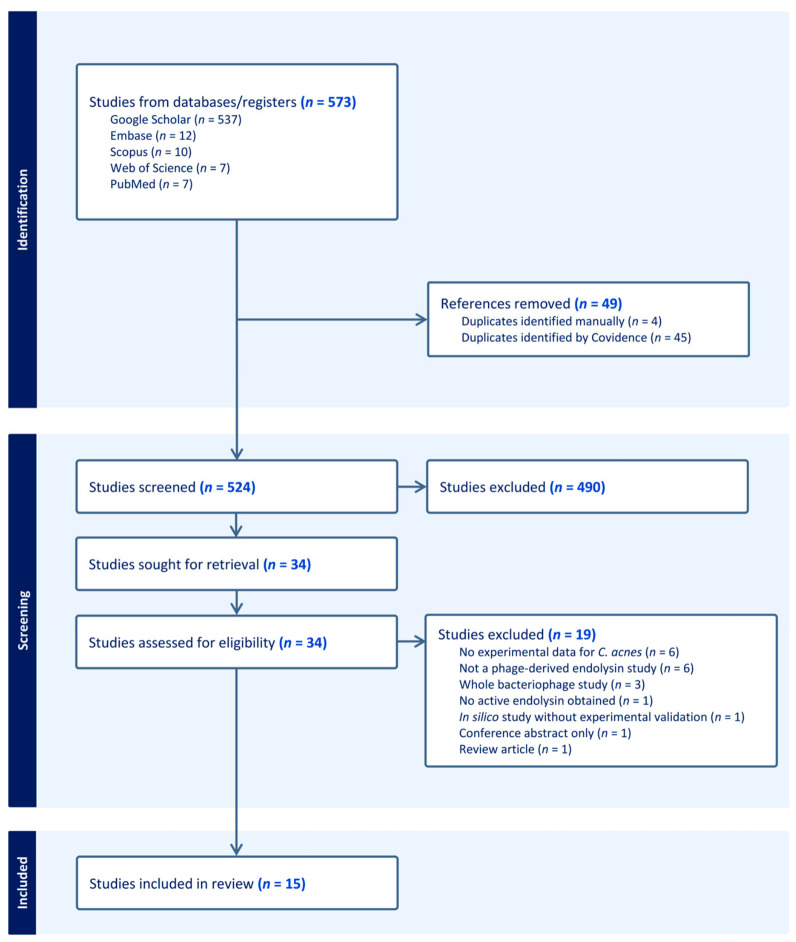
PRISMA flow diagram of the study selection process for the scoping review of phage-derived endolysins targeting *Cutibacterium acnes*.

Full-text assessment was performed based on pre-defined inclusion and exclusion criteria. Reasons for exclusion at the full-text evaluation stage were recorded and grouped, as presented in the PRISMA flow diagram (Figure 3).

### 4.3. Inclusion Criteria

Publications were eligible for inclusion in the review if they met all of the following criteria:

(1) Publication type: Original empirical studies (in vitro, ex vivo, in vivo), preclinical studies, patent applications describing the use of endolysins against *C. acnes*, and dissertations or theses containing primary experimental data.
(2) Investigated agents: Bacteriophage-derived endolysins (peptidoglycan hydrolases), recombinant or engineered endolysin variants, domain constructs (EAD and/or CBD), and peptides directly derived from phage endolysins.
(3) Target pathogen: Studies focusing on *C. acnes* (formerly *Propionibacterium acnes*); multi-species bacterial studies were eligible provided that separate data were reported for *C. acnes*.
(4) Scope of reported data: Publications were required to contain experimental data concerning at least one of the following aspects: mechanism of action, antimicrobial activity, specificity, impact on biofilm, or safety profile. For patents, an explicit description of the therapeutic application concept against *C. acnes* was required.
(5) Clinical context: Direct reference to acne vulgaris was not mandatory, provided that the study investigated *C. acnes* as a potential skin pathogen.

### 4.4. Exclusion Criteria

Publications were excluded if they met any of the following criteria:

(1) Studies focusing on whole bacteriophages without an analysis of the isolated endolysin.
(2) Studies on phage therapy lacking a lysin component.
(3) Studies on lytic enzymes not derived from bacteriophages.
(4) Studies based exclusively on computational modelling without experimental validation.
(5) Absence of experimental data directly relating to *C. acnes*.
(6) Lack of access to the full text of the publication.
(7) Secondary publications (e.g., review articles, commentaries, expert opinions).
(8) Full texts available only in languages other than English.

### 4.5. Data Extraction and Synthesis

Data extraction was performed using a pre-defined template encompassing the following variables:

(1) Author and year of publication.
(2) Type of lysin construct.
(3) Experimental model (in vitro, ex vivo, in vivo, biofilm model, skin models).
(4) Specificity of action.
(5) Type and scope of antimicrobial activity.
(6) Evaluation of anti-biofilm activity.
(7) Safety data.
(8) Stage of development (conceptual, preclinical, translational).

The results underwent narrative synthesis and were structured thematically.

In line with the standard methodology of scoping reviews, neither a formal evaluation of methodological quality nor a risk of bias assessment was conducted for the included studies.

## 5. Conclusions and Clinical Perspectives

Phage-derived endolysins represent a promising group of antimicrobial molecules for the treatment of acne vulgaris, particularly due to their enhanced selectivity towards *C. acnes* strains compared with conventional antimicrobial therapies. However, available data regarding their dermatological application remain largely limited to in vitro studies, while parameters related to pharmacokinetics, pharmacodynamics, formulation stability, and in vivo efficacy remain poorly characterised.

Significant translational challenges include not only preserving the antimicrobial activity of endolysins but also ensuring their effective delivery to the pilosebaceous unit and maintaining their enzymatic stability within the complex skin microenvironment. Further investigation into potential selectivity towards specific *C. acnes* phylotypes, anti-biofilm efficacy, and the long-term impact on the skin microbiome remains essential.

The future development of endolysin-based therapies will likely require the integration of advanced protein engineering strategies with modern drug delivery systems, alongside comprehensive translational evaluation using in vivo models and clinical trials.

## Figures and Tables

**Table 1:** Characteristics, specificity, and antimicrobial activity of endolysins and phage-derived constructs targeting *C. acnes*.

Author, Year	Lysin Type	Experimental Model and Development Stage	Specificity	Antimicrobial Activity	Safety Data
Kim et al., 2023 [22]	Recombinant full-length CAP10-3 endolysin	In vitro preclinical study	*C. acnes* (KCTC 3314); other species not evaluated	log CFU/mL: 6.15 → 4.65 (560 µg/mL, 8 h); OD_600_: 0.35 (175 µg/mL, 120 min); dose-dependent lytic activity	Not evaluated
Varotsou et al., 2023 [23]	Recombinant full-length endolysin (PaAmi1) and fusion variants	In vitro preclinical study	*C. acnes*, *S. aureus*, *S. epidermidis*; other tested Gram-positive and Gram-negative species	Reduction in turbidity of *C. acnes* cell suspension: PaAmi1 39.7 ± 2.6%; DS1PaAmi1 41.4 ± 3.5%; PA1PaAmi1 56.9 ± 0.2% (100 µg, 90 min); dose-dependent lytic activity of PaAmi1; fusion variants exhibited higher activity relative to PaAmi1; increased lytic activity of PaAmi1 in the presence of 5 mM Zn^2+^	Not evaluated
Lee et al., 2025 [24]	Recombinant full-length CAP10-3 endolysin and its domains: N-terminal (N1) and C-terminal (C1)	In vitro preclinical study	*C. acnes* (KCTC 3314, KCTC 3320); for the N1 domain: no significant activity against *S. aureus* (KCTC 3881) and weak activity against *S. epidermidis* (CJNU 0702) at high concentrations	Results for strain KCTC 3314: log CFU/mL: FL 9.44 → 8.49; N1 9.32 → 8.19; C1 9.34 → 8.76 (50 µg/mL, 3 h); OD_600_: FL 0.547; N1 0.395; C1 0.816 (50 µg/mL, 3 h); domain-dependent lytic effect (N1 > FL > C1); N1 domain exhibited dose-dependent lytic activity	Not evaluated
Hussain et al., 2025 [25]	Full-length endolysin; HEC-based endolysin gel	In vitro and clinical pilot study (topical application)	*C. acnes* (13 multidrug-resistant (MDR) isolates); other species not evaluated	MIC: 1 μg/mL; MBC: 4 μg/mL; complete decolonisation of MDR *C. acnes* in acne lesions treated with the HEC gel	No allergic reactions or reported adverse events
Sela et al., 2025 [26]	Sequence-modified cationic lysin-derived peptide (P156)	In vitro preclinical study	*C. acnes* (ATCC 6919, KPA171202, HSS B–F); *S. aureus* (8325, USA300, USA400)	>3-log kill in 1 min; >5-log kill in 10 min (25 µg/mL); ≥3-log kill against all tested *C. acnes* and *S. aureus* strains (5 µg/mL); activity maintained across pH 5.5–8.0, 20–40 °C and NaCl ≤ 50 mM; bactericidal activity preserved in the presence of retinoic acid	No haemolysis of hRBCs; no other cytotoxicity assays performed

## Data Availability

No new data were created or analysed in this study. Data sharing is not applicable to this article.

## Supplementary Materials

The following supporting information can be downloaded at: https://www.mdpi.com/article/10.3390/antibiotics15070697/s1. File S1: Preferred Reporting Items for Systematic reviews and Meta-Analyses extension for Scoping Reviews (PRISMA-ScR) Checklist.
